# Social advantage is a neuroprotector for cognitive outcome in children born extremely preterm

**DOI:** 10.1111/dmcn.70218

**Published:** 2026-02-28

**Authors:** Dieter Wolke

**Affiliations:** ^1^ Department of Psychology University of Warwick Coventry UK

## Abstract

**This commentary is on the original article by Hooper et al. on pages** 1551–1560 **of this issue.**

Survival rates of children born very preterm and extremely preterm have increased since the late 1980s and active treatment for ever smaller infants (born at 22 weeks or 23 weeks) is on the rise. Children born extremely preterm in any follow‐up study are born at a particular time in history (i.e. cohort) and experience the obstetric and neonatal treatments of the time. Has changed treatment and improved survival led to better outcomes in recent decades? A recent meta‐analysis that included 61 cohorts of children born very preterm found no indication of even the slightest trend of improvement of cognitive outcome in children assessed at 4 to 7 years of age born between the late 1970s and 2015.^1^

Thus, the results of the Extremely Low Gestational Age Newborn (ELGAN) study of children born extremely preterm (some 22–24 years ago and followed‐up in their cognitive development at 2 years, 10 years, and 15 years of age) are still relevant for current survivors.^2^ The authors investigated which neonatal factors are related to levels of cognitive functioning and growth of cognitive abilities in children born extremely preterm over time. There were large main effects of white matter injury (WMI) (−1.26 SD) and of social disadvantage. Each one‐point increase in the four‐points scale in social disadvantage was associated with a –0.30 SD decrease in level and cognitive growth. Thus, the main effect sizes of social disadvantage (comparing lowest to highest) and WMI were similar, as also previously reported in a Canadian cohort.^3^

Contrary to the Canadian study, the ELGAN study found additionally to the main effects of WMI and social disadvantage an interaction between the two. Social advantage was less effective in altering cognitive abilities in those with WMI compared to those without WMI. This may suggest that social factors require a plastic brain to be affected by social advantage.

Longitudinal studies of brain development indicate that the lower the gestation, the higher is extranormal development in more regions of the brain. However, brain development after preterm birth is highly heterogeneous in both severity and patterns of deviations.^4^ Most importantly, it has been shown that distal social variables such as higher socioeconomic status at birth and proximal social factors such as better parent–infant relationship in early infancy are positively associated with subsequent cortical thickness into adulthood (i.e. affecting neuroplasticity).^4^ The lower the gestational age, the relative larger were the effects of high socioeconomic status on cortical thickness development. Thus, looking at brain development such as cortical thickness directly, social factors provide a neuroprotective effect.

The combined evidence points to several implications. First, we now have overwhelming evidence that social factors such as low socioeconomic status or social disadvantage have similar adverse impact on long‐term cognitive outcome as brain injury or other severe neonatal complications. Second, while the search for pharmacological neuroprotective therapies continues, ‘… in the meantime, bundles of care protecting and nurturing the brain in the neonatal intensive care and beyond should be widely implemented in an effort to limit injury and promote neuroplasticity’.^5^ Third, distal social factors such as socioeconomic status are much more difficult to alter than proximal social factors such as parent–child interaction, including parental sensitivity. It is thus as important to record in detail social factors and care (e.g. family‐centred care) in the neonatal unit record system as it is to document medical neonatal complications. Furthermore, follow‐up for clinical or research purposes should assess family, parent–child, and peer interactions. These latter interactions show great promise to promote adaptive development and protect against adverse outcomes in children born preterm.

## Data Availability

Not required.
